# 

**Published:** 1855-09

**Authors:** 


VOL. II.] AUG. & SEPT., 1855. [NO. VI.

### IOWA

### MEDICAL JOURNAL.

CONDUCTED BY THE FACULTY OF THE

MEDICAL DEPARTMENT OF THE IOWA UNIVERSITY.

PUBLISHED EVERY ALTERNATE MONTH.

TERMS—TWO DOLLARS IN

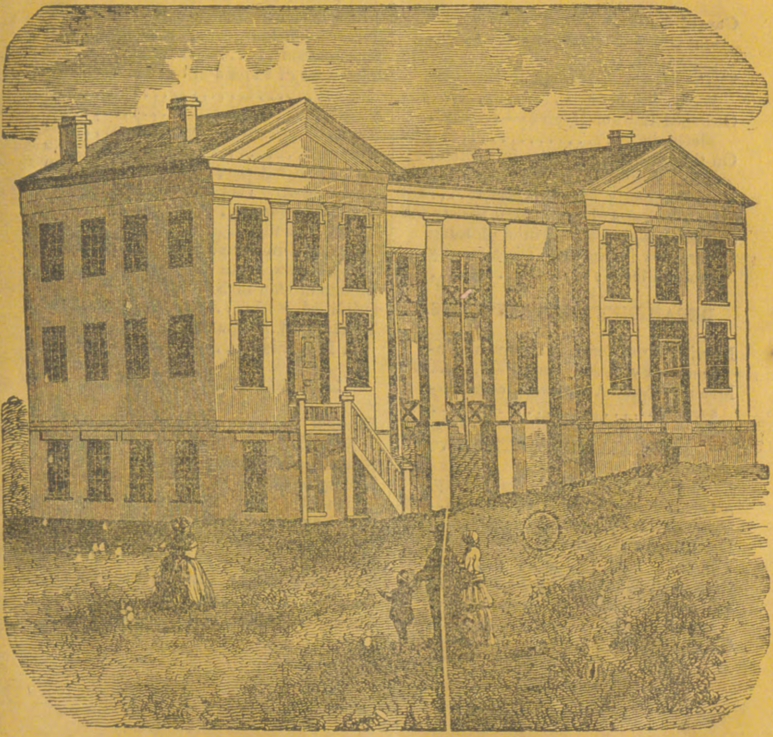

KEOKUK, IOWA:

PRINTED AT THE WHIG BOOK AND JOB OFFICE.

Address all Communications, post-paid, to "Medical College, Box 109, Keokuk, Iowa.

